# Short-term forecasting of daily COVID-19 cases in Brazil by using the Holt’s model

**DOI:** 10.1590/0037-8682-0283-2020

**Published:** 2020-06-03

**Authors:** Edson Zangiacomi Martinez, Davi Casale Aragon, Altacílio Aparecido Nunes

**Affiliations:** 1 Universidade de São Paulo, Faculdade de Medicina de Ribeirão Preto, Ribeirão Preto, SP, Brasil.

**Keywords:** COVID-19, Coronavirus disease, Forecasting, Statistical models, Epidemiology

## Abstract

**INTRODUCTION:**

We evaluated the performance of the Holt’s model to forecast the daily COVID-19 reported cases in Brazil and three Brazilian states.

**METHODS:**

We chose the date of the first COVID-19 case to April 25, 2020, as the training period, and April 26 to May 3, 2020, as the test period.

**RESULTS:**

The Holt’s model performed well in forecasting the cases in Brazil and in São Paulo and Minas Gerais states, but the forecasts were underestimated in Rio de Janeiro state.

**Conclusions:**

The Holt’s model can be an adequate short-term forecasting method if their assumptions are adequately verified and validated by experts.

Coronavirus disease (COVID-19) is caused by SARS-CoV-2 (or 2019-nCOV), a pathogen that primarily targets the human respiratory system^1^. The most common symptoms at the onset of the illness are cough, fever, and fatigue^2^. The first cases were reported in December 2019 in Wuhan, Hubei Province, China, and rapidly spread throughout the country and then the world. In January 2020, the World Health Organization (WHO) declared that COVID-19 is a “public-health emergency of international concern”^3^. 

To contribute in addressing the challenge of predicting the spread of the disease and obtaining short-term predictions, different types of mathematical and statistical models can be used (see ^4^ and ^5^ as examples). Accordingly, let us to consider an epidemic curve as a time series data of the daily number of cases of a disease, and let *Y*_*t*_ be the cumulative number of confirmed cases on day *t*. It is expected that this curve initially grows exponentially, but at a given moment, it slows and approaches a limit. Therefore, the simple exponential model is commonly used to describe the initial phase of an outbreak^6^, and S-shaped models such as the logistic, Gompertz, log-normal, and Richards models are widely used to model all the reported cumulative cases of a disease^7^. In the present communication, we alternatively propose the use of double exponential smoothing for short-term forecasting of the daily COVID-19 cases in Brazil, before the peak of the cases.

Methods based on exponential smoothing are often used for forecasting. These methods are based on a moving average of past values only, so that the smoothed value at the present time is used as the forecast of the next value^8^. The Holt-Winters exponential model is a more general method for smoothing the data when trend and seasonality are present. The double exponential smoothing (also called the Holt's method) is a special case in which seasonality is absent. Finally, the single exponential smoothing is used when no trend or seasonal components are present. In the equation for the Holt’s method, the forecasted value of the series at time t is given by

$${\hat{Y}}_{t}=L_{t-1}+T_{t-1}$$

where *L*_*t*_ is the estimated level given by

$$L_{t}={\alpha Y}_{t}+\left(1-\alpha \right)\left(L_{t-1}+T_{t-1}\right),$$

*T*_*t*_ is the estimated slope given by

$$T_{t}={\beta (L}_{t}-L_{t-1})+\left(1-\beta \right)T_{t-1},$$

and α and β are the smoothing parameters (technical details can be found in ^8^). For applying the Holt's model, we used the *holt* function in the *forecast* library of the R language (version 3.6.2).

Data on daily COVID-19 cases were obtained from the Brazilian Health Ministry (available at https://covid.saude.gov.br/). Our analysis included data from the whole country and from the Brazilian states of São Paulo, Minas Gerais, and Rio de Janeiro. These are the three most populous Brazilian states, and together, have more than 80 million inhabitants (approximately 40% of the Brazilian population). We considered the daily reports from the date on which the first case was notified in Brazil and in each state up to April 25, 2020, as the training period. The values of the validation period were the correspondent observations from April 26 to May 3, 2020. We compared the forecast accuracy of the Holt's method with those obtained by fitting the traditional logistic, Gompertz, log-normal, and Richards growth curves. These comparisons were based on the mean absolute percent error (MAPE), a measure based on the differences between the forecasted and the actual values. The Theil's U entropy coefficient was used as a measure of out-of-sample forecasting accuracy^9^. When this coefficient is higher than 1, the forecasts under consideration are less accurate than those offered via a naïve approach, i.e., a simple method in which the forecasts are equal to the last observed value.

Figure 1 shows the cumulative number of reports of COVID-19 until April 25, 2020, in Brazil and in the states of São Paulo, Minas Gerais, and Rio de Janeiro, and the forecasted values from the Holt’s method with their correspondent prediction intervals. These values are detailed in Table 1, which also compares the actual and forecasted daily values from April 26 to May 3, 2020. The Theil's U coefficients are lower than 1 for the forecasts considering the data from Brazil and the states of São Paulo and Minas Gerais, but higher than 1 when the data from the state of Rio de Janeiro is considered (values are shown in Figure 1). In addition, as observed in Table 1, almost all the actual daily reports of COVID-19 belong to the correspondent 95% prediction intervals, except for the forecasts considering the data from the state of Rio de Janeiro. The estimated number of cases tends to underestimate the actual reports of COVID-19 from April 27, owing to a sudden increase in notifications that started on this date.

**FIGURE 1:**
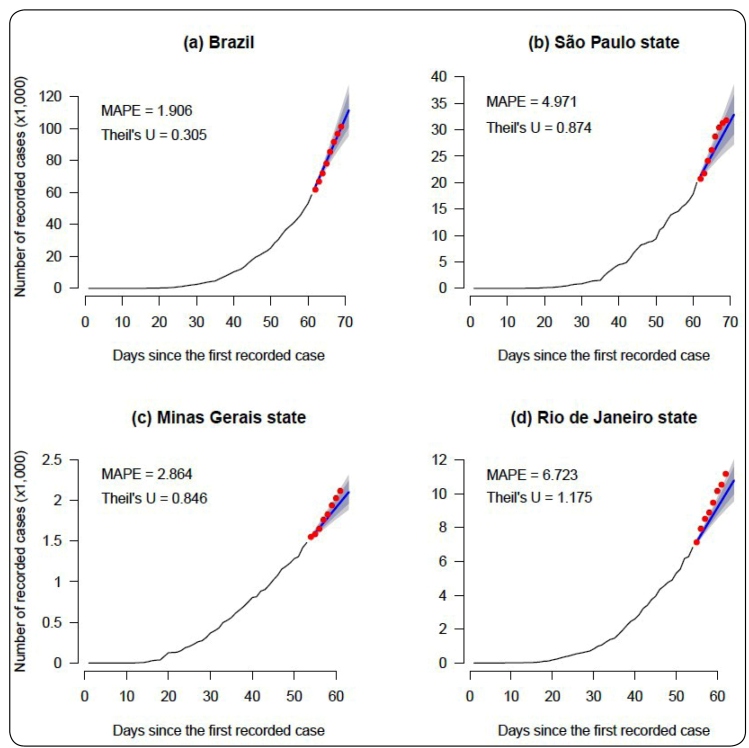
Time series for **(a)** Brazil and the states of **(b)** São Paulo, **(c)** Minas Gerais, and **(d)** Rio de Janeiro, showing point forecasts and 80% and 95% prediction intervals obtained using the Holt’s model (represented by the dark gray and the clear gray areas, respectively). The red points represent the actual number of notified cases.

**TABLE 1:** Daily COVID-19 cases and the correspondents forecasts from the Holt’s method (with 95% prediction intervals), from April 26 to May 3, 2020.

	Day	Observed	Forecasted	95% prediction
		values	values	interval
				
**Brazi**l	April 26	61888	63598.77	62684.76-64512.78
	April 27	66501	68898.82	67035.24-70762.39
	April 28	71886	74198.86	71131.73-77265.99
	April 29	78162	79498.91	75031.55-83966.26
	April 30	85380	84798.95	78762.27-90835.64
	May 1	91589	90099.00	82341.48-97856.51
	May 2	96559	95399.05	85781.96-105016.13
	May 3	101147	100699.09	89093.56-112304.62
				
**São Paulo state**	April 26	20715	21288.91	20630.67-21947.16
	April 27	21696	22573.96	21473.87-23674.05
	April 28	24041	23859.01	22300.00-25418.02
	April 29	26158	25144.06	23096.16-27191.96
	April 30	28698	26429.11	23860.05-28998.16
	May 1	30374	27714.16	24591.88-30836.44
	May 2	31174	28999.21	25292.55-32705.87
	May 3	31772	30284.26	25963.17-34605.35
				
**Minas Gerais state**	April 26	1548	1537.80	1501.70-1573.89
	April 27	1586	1600.34	1550.50-1650.19
	April 28	1649	1662.89	1597.30-1728.48
	April 29	1758	1725.44	1642.42-1808.47
	April 30	1827	1787.99	1686.04-1889.94
	May 1	1935	1850.54	1728.31-1972.77
	May 2	2023	1913.09	1769.32-2056.86
	May 3	2118	1975.64	1809.15-2142.13
				
**Rio de Janeiro state**	April 26	7111	7169.33	7001.87-7336.78
	April 27	7944	7570.99	7345.19-7796.80
	April 28	8504	7972.66	7665.42-8279.90
	April 29	8869	8374.33	7968.45-8780.20
	April 30	9453	8775.99	8257.85-9294.13
	May 1	10166	9177.66	8535.73-9819.59
	May 2	10546	9579.32	8803.43-10355.22
	May 3	11139	9980.99	9061.89-10900.10

Considering the data from Brazil, the MAPE values for the forecasting methods based on the logistic, Gompertz, log-normal, and Richards curves are 17.09, 10.84, 9.05, and 10.84, respectively. These corresponding values are 21.81, 15.70, 14.37, and 15.70 considering the data from the state of São Paulo; 14.63, 8.52, 5.13, and 8.52 considering the data from the state of Minas Gerais; and 18.00, 10.54, 8.18, and 10.55 considering the data from the state of Rio de Janeiro. In all the situations, the MAPE values for the forecast based on the Holt’s method (shown in Figure 1) are smaller than those obtained from the fit of the traditional growth curves, showing a better performance of the Holt’s method compared to the others (even for the forecasts using data from the state of Rio de Janeiro). Figure 2 provides a visual comparison between the actual daily reports of COVID-19 from April 26 to May 3, 2020, and the forecasts from the different methods. Exponential models were not used in this analysis, as they performed poorly in describing the epidemic curves based on the training period. 

**FIGURE 2:**
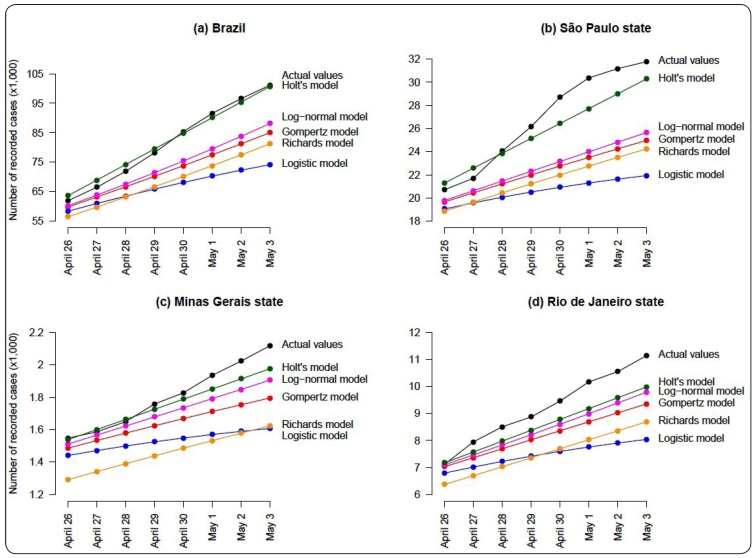
Comparison between the actual number of notified cases of COVID-19 and the forecasted values obtained from the Holt’s, logistic, Gompertz, log-normal, and Richards models, for the period from April 26 to May 3, 2020, considering **(a)** Brazil and the states of **(b)** São Paulo, **(c)** Minas Gerais, and **(d)** Rio de Janeiro.

In order to correctly interpret the results of these statistical models, we should keep in mind an important quote from Saffo^10^: “*The goal of forecasting is not to predict the future but to tell you what you need to know to take meaningful action in the present*”. In this sense, the out-of-sample predicted values should be seen primarily as the daily number of cases of COVID-19 that we would expect to find if the epidemic curve continues to grow with the same behavior observed during the training period. The volatility of the time series of reported cases is highly dependent on extrinsic factors (such as the availability of tests for essential screening) as well as in the speed of updating and the availability of results and changes in the mitigation measures^11^. In turn, these factors are affected by the incubation period of the virus of approximately 14 days (interquartile range, 8-17 days), with variations according to the age of the patient and status of the patient's immune system^12^. Therefore, we can conclude that the Holt’s model showed good forecast performance for the data from Brazil and the states of São Paulo and Minas Gerais, probably because the behavior of the epidemic curves do not change significantly at the beginning of the validation period. This did not happen considering the data from the state of Rio de Janeiro. However, we do not believe that this is a defect of the method but rather a failure to comply with its assumptions. These observations apply to any mathematical or statistical model used for obtaining predictions of cases of COVID-19, and for that reason, every forecasting model should be accompanied by the expertise of trained individuals familiar with the dynamics of infectious diseases. In addition, we reinforce that the generalization of the results of this study is restricted to the objective of obtaining short-term forecasts for the cumulative number of cases of COVID-19 in a determined population, as the Holt’s model has a low sensitivity for predicting the peak of the outbreak or for providing long-term forecasts.

An important and obvious limitation of this study is that it was conducted only using the reported number of COVID-19 cases that have been officially notified. Considering the insufficient number of screening tests and the consequent low effectiveness in confirming cases of COVID-19 in Brazil, it is obviously expected that the actual number of cases of the disease is much greater than that presented here^13^ ^,^ ^14^. Nevertheless, while these data are biased, they are the only source of information available that can guide our efforts to understand the outbreak dynamics. Because of the urgency for information that can be useful for the decision-making processes during the course of an epidemic, we consider that these data are “that's what we have for today,” and that they can be properly used when their potential limits are well discussed. As an additional commentary, the models presented in this study only represent the cumulative number of cases of a disease, while other more complex models can provide more accurate predictions by also taking into account the number of susceptible and recovered individuals (called susceptible-infected-recovered [SIR] models and their extensions)^7^.

In conclusion, despite all the problems described herein that make the prediction of cases of COVID-19 a challenging task, the Holt’s model can be an adequate alternative to the traditional S-shaped curves if their assumptions are adequately verified and validated by experts.

## References

[1] Rothan HA, Byrareddy SN (2020). The epidemiology and pathogenesis of coronavirus disease (COVID-19) outbreak. J Autoimmun.

[2] Huang C, Wang Y, Li X, Ren L, Zhao J, Hu Y (2020). Clinical features of patients infected with 2019 novel coronavirus in Wuhan, China. Lancet.

[3] Wu D, Wu T, Liu Q, Yang Z (2020). The SARS-CoV-2 outbreak: what we know. Int J Infect Dis.

[4] Tuite AR, Fisman DN (2020). Reporting, epidemic growth, and reproduction numbers for the 2019 novel coronavirus (2019-nCoV) epidemic. Ann Intern Med.

[5] Kucharski AJ, Russell TW, Diamond C, Liu Y, Edmunds J, Funk S (2020). Early dynamics of transmission and control of COVID-19: a mathematical modelling study. Lancet Infect Dis.

[6] Ma J, Dushoff J, Bolker BM, Earn DJ (2014). Estimating initial epidemic growth rates. Bull Math Biol.

[7] Brauer F, Castillo-Chavez C, Feng Z (2019). Mathematical Models in Epidemiology.

[8] Peter J. Brockwell, Richard A (2016). Davis. Introduction to Time Series and Forecasting.

[9] Theil H (1971). Applied economic forecasting.

[10] Saffo P (2007). Six rules for effective forecasting. Harv Bus Rev.

[11] Anderson RM, Heesterbeek H, Klinkenberg D, Hollingsworth TD (2020). How will country-based mitigation measures influence the course of the COVID-19 epidemic?. Lancet.

[12] Tay MZ, Poh CM, Rénia L, MacAry PA, Ng LFP (2020). The trinity of COVID-19: immunity, inflammation and intervention. Nat Rev Immunol.

[13] Krantz SG, Rao ASRS (2020). Level of under-reporting including under-diagnosis before the first peak of COVID-19 in various countries: Preliminary Retrospective Results Based on Wavelets and Deterministic Modeling. Infect Control Hosp Epidemiol.

[14] Werneck GL, Carvalho MS (2020). A pandemia de COVID-19 no Brasil: crônica de uma crise sanitária anunciada. Cad. Saúde Pública.

